# The potential of systemic immune-inflammation index in predicting outcomes of facial palsy in patients with Ramsay Hunt syndrome treated by acupuncture

**DOI:** 10.3389/fneur.2025.1640058

**Published:** 2025-09-25

**Authors:** Yinglin Fang, Xiaoping Zhong, Jing Zuo, Jianlong Wang

**Affiliations:** ^1^Department of Acupuncture and Moxibustion, Hangzhou Xiaoshan Hospital of Traditional Chinese Medicine, Hangzhou, Zhejiang, China; ^2^Department of Rehabilitation, Affiliated Xiaoshan Hospital, Hangzhou Normal University, Hangzhou, Zhejiang, China; ^3^Department of Ultrasound, Hangzhou Xiaoshan Hospital of Traditional Chinese Medicine, Hangzhou, Zhejiang, China; ^4^Department of Neurology, Hangzhou Xiaoshan Hospital of Traditional Chinese Medicine, Hangzhou, Zhejiang, China

**Keywords:** Ramsay Hunt syndrome, facial palsy, acupuncture, systemic immune-inflammation index, outcome

## Abstract

**Objective:**

To investigate the association between the systemic immune-inflammation index (SII) and treatment outcomes of facial palsy in patients with Ramsay Hunt syndrome (RHS) receiving acupuncture therapy.

**Methods:**

This retrospective observational study enrolled 125 adult patients diagnosed with RHS and treated with acupuncture at Hangzhou Xiaoshan Hospital of Traditional Chinese Medicine. Inclusion required clinical confirmation of RHS, baseline House–Brackmann (H-B) grade IV–VI facial palsy, and symptom onset within 7 days. Patients were categorized into effective (*n* = 85) and ineffective (*n* = 40) groups based on at least one-grade improvement in H-B score post-treatment. Baseline clinical and hematological parameters, including neutrophil, lymphocyte, and platelet counts, were collected to calculate SII: (platelet count × neutrophil count) / lymphocyte count. Statistical analyses included univariate and multivariate logistic regression, as well as receiver operating characteristic (ROC) curve analysis to assess the associative performance of SII and BMI.

**Results:**

Compared with the effective group, the ineffective group exhibited significantly elevated neutrophil counts and SII, and reduced lymphocyte counts (all *p* < 0.05). Multivariate logistic regression identified SII as an independent factor associated with reduced likelihood of acupuncture efficacy (adjusted OR = 0.997, 95% CI: 0.995–0.998, *p* < 0.001), while BMI lost significance after adjustment. ROC analysis demonstrated a moderate discriminative ability of SII (AUC = 0.839, 95% CI: 0.765–0.913), with optimal sensitivity (70.0%) and specificity (89.4%) at a cut-off value of 1262.935. A combined model of SII and BMI showed only a marginal change in discriminative performance (AUC = 0.840, sensitivity = 85.9%, specificity = 72.5%).

**Conclusion:**

SII is an independent biomarker associated with the clinical efficacy of acupuncture in patients with RHS-related facial palsy. Incorporating SII into clinical assessments may contribute to improved prognostic evaluation and support individualized therapeutic strategies.

## Introduction

Ramsay Hunt syndrome (RHS), also referred to as herpes zoster oticus, is a neurotropic viral disorder caused by the reactivation of latent varicella-zoster virus (VZV) within the geniculate ganglion of the facial nerve (1, 2). Clinically, RHS is characterized by the sudden onset of unilateral peripheral facial nerve palsy, accompanied by vesicular eruptions in the auricular region and external auditory canal, often coupled with otologic symptoms such as otalgia, tinnitus, vertigo, and varying degrees of sensorineural hearing loss (1, 2). These features are thought to reflect the neurotropic and neuroinflammatory effects of VZV, which may extend beyond the facial nerve to involve the vestibulocochlear nerve, contributing to a broader spectrum of cranial neuropathies (3).

RHS accounts for approximately 12% of all cases of peripheral facial paralysis, making it the second most common etiology after Bell’s palsy (4, 5). Notably, patients with RHS tend to experience more severe initial facial nerve dysfunction, slower recovery, and a higher incidence of long-term sequelae compared to those with idiopathic facial palsy (4, 5). Despite the early administration of antiviral agents (e.g., acyclovir or valacyclovir) in combination with corticosteroids, which remains the mainstay of conventional pharmacologic treatment, clinical outcomes remain suboptimal in a substantial subset of patients (6, 7). Persistent facial asymmetry, synkinesis, and functional impairment continue to affect quality of life, highlighting the urgent need for adjunctive therapeutic interventions and objective biomarkers capable of guiding individualized treatment planning and prognostication.

Acupuncture, rooted in traditional Chinese medicine, has been increasingly employed as a complementary therapeutic modality for facial nerve palsy, including both Bell’s palsy and RHS. Its proposed mechanisms involve the regulation of neurovascular dynamics, attenuation of neuroinflammation, promotion of axonal regeneration, and modulation of peripheral and central nervous system plasticity through specific acupoint stimulation (8–10). Clinical evidence suggests that acupuncture may facilitate faster and more complete recovery of facial nerve function, particularly when initiated during the acute or subacute phase of paralysis (11–13). However, the therapeutic response to acupuncture is highly variable across individuals, and the absence of validated predictive markers continues to impede optimal patient selection and outcome forecasting.

In this context, the systemic immune-inflammation index (SII), calculated as platelet count × neutrophil count / lymphocyte count, has emerged as a promising, readily accessible hematological biomarker that integrates key components of innate immunity, systemic inflammation, and immune suppression (14–16). A growing body of evidence links elevated SII values with unfavorable prognosis in a range of pathological conditions, including systemic inflammatory diseases (17), autoimmune disorders (16), cardiovascular events (18), and cerebrovascular insults (19). The SII is believed to reflect the complex interplay between pro-inflammatory neutrophilia, thrombocytosis associated with vascular injury and repair, and lymphopenia indicative of immune dysregulation.

Despite its expanding application in prognostic modeling across various medical disciplines, the relevance of SII in peripheral facial nerve disorders, particularly in RHS patients undergoing acupuncture therapy, has not yet been systematically explored. Therefore, this study aims to assess the association of SII with therapeutic outcomes of acupuncture in patients with RHS-induced facial palsy.

## Methods

### Participants enrollment

This retrospective observational study included patients diagnosed with RHS who received acupuncture therapy for peripheral facial paralysis at Hangzhou Xiaoshan Hospital of Traditional Chinese Medicine from January 2022 to June 2024. Patients were considered eligible for inclusion if they met all of the following criteria: (1) a clinical diagnosis of RHS characterized by acute unilateral peripheral facial nerve palsy in conjunction with typical herpetic vesicular eruptions in the auricular region and/or associated otologic symptoms (e.g., otalgia, vertigo, or hearing loss), with facial nerve dysfunction severity classified as House–Brackmann (H-B) grade IV, V, or VI at baseline, as patients with mild to moderate palsy (H-B grade II–III) usually achieve favorable spontaneous recovery under standard therapy, whereas severe cases carry a poorer prognosis and therefore provide a more appropriate population to evaluate the potential therapeutic contribution of acupuncture and the prognostic relevance of SII; (2) age ≥18 years; (3) onset of symptoms within 7 days prior to initiation of acupuncture treatment; (4) no prior surgical or invasive intervention for facial paralysis; and (5) availability of complete clinical records, laboratory data, and follow-up information. Patients were excluded if they met any of the following criteria: (1) facial palsy due to etiologies other than RHS (e.g., Bell’s palsy, cerebrovascular accident, trauma); (2) presence of central nervous system diseases or severe systemic comorbidities such as autoimmune disorders or malignancy; (3) receipt of immunosuppressive or cytotoxic therapy within 3 months prior to enrollment; or (4) incomplete data or loss to follow-up. The ethical approval for this study was reviewed and formally waived by the Institutional Ethics Committee of Hangzhou Xiaoshan Hospital of Traditional Chinese Medicine because it was retrospective in design, based solely on anonymized clinical and laboratory data from existing medical records, and involved no direct patient contact or additional interventions. All procedures were conducted in accordance with the principles outlined in the Declaration of Helsinki, and written informed consent for the use of medical records in research had been obtained from all patients at the time of initial treatment, with confidentiality strictly maintained during data handling and analysis.

All enrolled patients received standardized acupuncture treatment following their initial diagnosis and were subsequently evaluated for clinical improvement in facial nerve function. Clinical efficacy was assessed using the H-B grading system. The primary outcome was defined as an improvement of at least one H-B grade following the acupuncture treatment course. Patients who met this criterion were classified into the effective group (*n* = 85), while those who showed no improvement were categorized into the ineffective group (*n* = 40).

### Acupuncture procedures

All patients received standardized acupuncture treatment performed by licensed acupuncturists with professional experience in the management of peripheral facial paralysis. The acupuncture protocol was based on traditional Chinese medicine theory and focused on restoring facial nerve function through both local and distal acupoint stimulation. The following acupoints were used in all patients: Yingxiang (LI20), Dicang (ST4), Jiache (ST6), Jiachengjiang (extra point), bilateral Hegu (LI4), Yifeng (TE17), Yangbai (GB14), Tongziliao (GB1), and Quanliao (SI18). These points were selected for their anatomical correlation with the facial nerve branches and their traditional use in promoting facial muscle recovery, clearing wind, and activating local circulation. Sterile disposable acupuncture needles (Jiajian brand, 0.30 mm × 25 mm) were used. For patients who began treatment within 1 week of symptom onset, needles were inserted to a depth of approximately 3–5 mm using a balanced reinforcing-reducing technique (Pingbu Pingxie), without excessive stimulation. For patients starting acupuncture after 1 week, slightly stronger manual stimulation was applied after insertion to enhance therapeutic response. Each session lasted 30 min and was administered once daily. A total of 10 sessions constituted one treatment course, with a 5-day interval between courses as appropriate. All procedures were performed in a controlled environment under sterile conditions.

### Collection of clinical data

Clinical and laboratory data were collected from medical records at the time of patient enrollment. Baseline data were obtained prior to the initiation of acupuncture treatment and included both demographic and disease-related variables. Demographic parameters comprised age, sex, and body mass index (BMI). Clinical characteristics included smoking status (current, former, never), alcohol consumption (yes/no), duration from symptom onset to acupuncture initiation (days), laterality of facial palsy (left/right), presence of vertigo, hearing loss, vesicular rash, and history of antiviral and corticosteroid use. The severity of facial paralysis at baseline was evaluated using the House–Brackmann (H-B) facial nerve grading system. All grading assessments were performed by trained clinicians with experience in facial nerve disorders. Hematological parameters were measured from peripheral venous blood samples collected within 24 h before the first acupuncture session, including Neutrophil count (×10^9^/L), Lymphocyte count (×10^9^/L) and Platelet count (×10^9^/L).

### Calculation of SII

The SII was calculated to assess the balance between inflammatory and immune status using peripheral blood parameters obtained within 24 h prior to acupuncture initiation. Blood counts were measured with automated hematology analyzers. SII was defined as: SII = (Platelet count × Neutrophil count) / Lymphocyte count, with all values expressed in ×10^9^/L. All laboratory data were verified for accuracy; implausible values were excluded after source confirmation.

### Statistical analysis

All statistical analyses were performed using SPSS version 21 (IBM Corp., Armonk, NY, USA) and MedCalc version 20.0 (MedCalc Software Ltd., Ostend, Belgium). The Shapiro–Wilk test was utilized to test normality. Using mean ± standard deviation (SD), data which showed a normal distribution were described, and the independent samples *t*-test was used for their comparison between groups. For non-normally distributed variables, median (interquartile range) was used for description and Mann–Whitney U test was utilized for their comparisons between groups. Categorical variables were presented as frequencies and percentages and compared using the chi-square test or Fisher’s exact test, as appropriate. To explore potential factors associated with clinical efficacy, univariate logistic regression analysis was first performed, and variables with *p* < 0.05 were subsequently included in the multivariate logistic regression model using a forward stepwise approach. Results were reported as odds ratios (ORs) with 95% confidence intervals (CIs). Multicollinearity was assessed before model inclusion. Receiver operating characteristic (ROC) curve analysis was used to assess the performance of SII. The area under the ROC curve (AUC) was calculated for evaluation of discrimination, and the Youden index was used to determine the optimal cut-off value, with the threshold corresponding to the maximum Youden index selected as the best balance between sensitivity and specificity. A two-tailed *p*-value < 0.05 was considered statistically significant.

## Results

### Baseline features between responders and non-responders

The baseline features of patients in the effective and ineffective groups are presented in Table 1. A total of 125 patients diagnosed with RHS and treated with acupuncture were included in this study, among whom 85 were categorized as the effective group and 40 as the ineffective group based on clinical improvement in facial nerve function. No significant differences were observed in age between the responders and non-responders (55.88 ± 4.77 vs. 56.55 ± 4.53 years, *p* = 0.460). Gender distribution was also similar (*p* = 0.701), with females representing 55.29% of the responders and 50.00% of the non-responders. Smoking status (*p* = 0.381), alcohol consumption (*p* = 0.683), and time from symptom onset to initiation of treatment (5.12 ± 1.48 vs. 5.48 ± 1.26 days, *p* = 0.189) did not differ significantly between groups. The severity of facial palsy at baseline, as assessed by the H-B grading system, was comparable across groups (*p* = 0.694), with Grade IV constituting the largest proportion in both the responders (52.94%) and non-responders (45.00%). Similarly, there were no statistically significant differences in the presence of vertigo (*p* = 0.346), hearing loss (*p* = 0.541), vesicular rash (*p* = 0.115), side of facial involvement (*p* = 0.446), prior antiviral use (*p* = 0.959), or concomitant corticosteroid administration (*p* = 0.186). Nevertheless, BMI was lower in the responders compared to the non-responders (23.89 ± 2.19 vs. 25.14 ± 2.34 kg/m^2^, *p* < 0.05). Analysis of inflammatory markers revealed marked group differences. Neutrophil count was significantly higher in the non-responders (6.16 ± 0.93 vs. 3.76 ± 1.00 × 10^9^/L, *p* < 0.05), while lymphocyte count was significantly lower (1.26 ± 0.33 vs. 1.69 ± 0.30 × 10^9^/L, *p* < 0.05). Platelet count of the two groups was not significantly different (258.95 ± 50.93 vs. 245.46 ± 41.36 × 10^9^/L, *p* = 0.117). Importantly, the SII was significantly elevated in the non-responders compared to the responders (1326.36 ± 390.12 vs. 784.20 ± 375.44, *p* < 0.05). These findings suggest that elevated SII may be associated with reduced therapeutic responsiveness to acupuncture in RHS patients and could serve as a potential indicator associated with clinical outcomes in this population.

**Table 1:** Baseline characteristics of the ineffective and effective groups.

Indices	Ineffective group (*n* = 40)	Effective group (*n* = 85)	*p*
Age (years)	56.55 ± 4.53	55.88 ± 4.77	0.46
Sex [*n* (%)]			0.701
Male	20 (50.00)	38 (44.71)	
Female	20 (50.00)	47 (55.29)	
BMI (kg/m^2^)	25.14 ± 2.34	23.89 ± 2.19	<0.05
Smoking status [*n* (%)]			0.381
Current	14 (35.00)	27 (31.76)	
Former	11 (27.50)	16 (18.82)	
Never	15 (37.50)	42 (49.41)	
Alcohol consumption [*n* (%)]			0.683
Yes	14 (35.00)	26 (30.59)	
No	26 (65.00)	59 (69.41)	
Duration from onset to treatment (days)	5.48 ± 1.26	5.12 ± 1.48	0.189
H-B grade [*n* (%)]			0.694
IV	18 (45.00)	45 (52.94)	
V	13 (32.50)	23 (27.06)	
VI	9 (22.50)	17 (20.00)	
Symptoms & Signs [*n* (%)]			
Vertigo	23 (57.50)	41 (48.24)	0.346
Hearing loss	14 (35.00)	25 (29.41)	0.541
Vesicular rash	37 (92.50)	68 (80.00)	0.115
Affected side [*n* (%)]			0.446
Left	23 (57.50)	42 (49.41)	
Right	17 (42.50)	43 (50.59)	
Prior antiviral [*n* (%)]			0.959
Yes	29 (72.50)	62 (72.94)	
No	11 (27.50)	23 (27.06)	
Concomitant corticosteroid use [*n* (%)]			0.186
Yes	24 (60.00)	45 (52.94)	
No	16 (40.00)	40 (47.06)	
Laboratory tests			
Neutrophil count (×10^9^/L)	6.16 ± 0.93	3.76 ± 1.00	<0.05
Lymphocyte count (×10^9^/L)	1.26 ± 0.33	1.69 ± 0.30	<0.05
Platelet count (×10^9^/L)	258.95 ± 50.93	245.46 ± 41.36	0.117
SII	1326.36 ± 390.12	784.20 ± 375.44	<0.05

### Uni- and multi-variate logistic regression analysis exploring factors associated with the clinical efficacy of acupuncture in the facial palsy of Ramsay-Hunt syndrome patients

To explore the determinants of clinical effectiveness of acupuncture in patients with RHS–induced facial paralysis, we conducted both univariate and multivariate logistic regression analyses. The univariate analysis identified several clinical and inflammatory parameters that were significantly associated with therapeutic outcomes. As presented in Table 2, BMI, neutrophil count, lymphocyte count, and the SII were found to be statistically significant. In particular, BMI demonstrated a negative correlation with treatment efficacy (*β* = −0.25, SE = 0.09, Wald *χ*^2^ = 7.65, *p* = 0.006), corresponding to an OR of 0.779 (95% CI, 0.652–0.930), indicating that patients with higher BMI were less likely to achieve favorable clinical response. Neutrophil count also exhibited a significant inverse association (*β* = −2.387, SE = 0.432, Wald *χ*^2^ = 30.518, *p* < 0.001), with an OR of 0.092 (95% CI, 0.039–0.214), suggesting that elevated neutrophil levels markedly reduced the possibility of clinical improvement. In contrast, lymphocyte count was positively and strongly associated with treatment success (*β* = 4.198, SE = 0.814, Wald *χ*^2^ = 26.577, *p* < 0.001), with an OR of 66.523 (95% CI, 13.487–328.123), highlighting its potential as a strong positive predictor. Additionally, SII was significantly and negatively associated with treatment outcome (*β* = −0.003, SE = 0.001, Wald *χ*^2^ = 28.135, *p* < 0.001), with an OR of 0.997 (95% CI, 0.995–0.998), suggesting that a higher systemic inflammatory burden could impair the efficacy of acupuncture therapy.

**Table 2:** The predicting factors on the clinical efficacy of acupuncture in the facial palsy of Ramsay-Hunt syndrome patients determined by univariate regression analysis.

Clinical indices	ß	SE	Wald *χ*^2^	*p*	OR	95% CI
BMI	−0.25	0.09	7.65	0.006	0.779	0.652 ~ 0.930
Neutrophil	−2.387	0.432	30.518	<0.001	0.092	0.039 ~ 0.214
Lymphocyte	4.198	0.814	26.577	<0.001	66.523	13.487 ~ 328.123
SII	−0.003	0.001	28.135	<0.001	0.997	0.995 ~ 0.998

To further elucidate which of these variables were independently associated with clinical efficacy, BMI and SII were included in a multivariate logistic regression analysis, as neutrophil and lymphocyte counts were excluded due to their direct involvement in the calculation of SII and strong collinearity with it, which could compromise model accuracy and stability. SII remained independently associated with treatment efficacy (Table 3). In the multivariate model, SII retained its statistical significance (*β* = −0.003, SE = 0.001, Wald *χ*^2^ = 24.063, *p* < 0.001), with an adjusted OR of 0.997 (95% CI, 0.995–0.998), supporting its role as a significant negative correlate. Conversely, the association between BMI and clinical outcome was no longer significant after adjusting for confounding variables (*β* = −0.077, SE = 0.111, Wald *χ*^2^ = 0.491, *p* = 0.483). Collectively, SII appears to be a potential independent biomarker associated with therapeutic outcomes following acupuncture intervention in this patient population.

**Table 3:** The predicting factors on the clinical efficacy of acupuncture in the facial palsy of Ramsay-Hunt syndrome patients determined by multivariate regression analysis.

Clinical indices	ß	SE	Wald *χ*^2^	*p*	OR	95% CI
BMI	−0.077	0.111	0.491	0.483	0.925	0.745 ~ 1.149
SII	−0.003	0.001	24.063	<0.001	0.997	0.995 ~ 0.998

### The ROC analysis assessing the association of SII with clinical efficacy of acupuncture for facial palsy in Ramsay-Hunt syndrome

**Figure 1:**
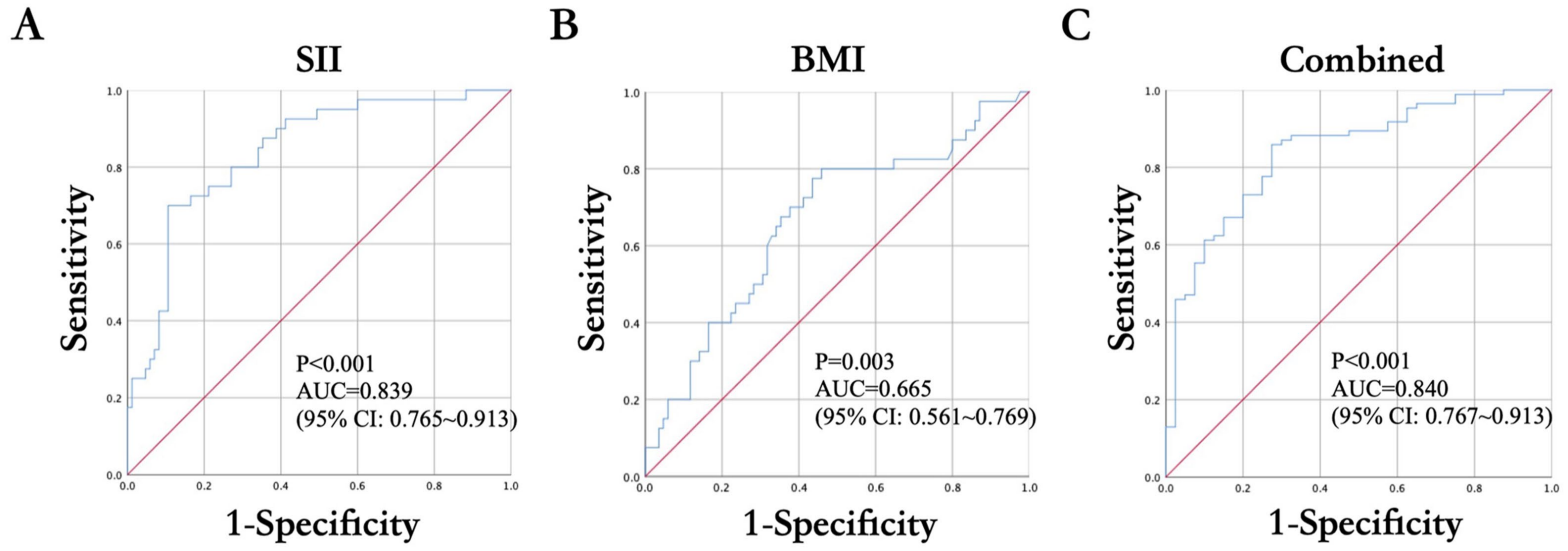
ROC curve evaluating the predicting effects of **(A)** SII, **(B)** BMI, **(C)** combined analysis on the clinical efficacy of acupuncture for facial palsy in Ramsay-Hunt syndrome patients. The blue line represents the true performance of BMI, while the red diagonal line indicates the reference line of random classification.

To further assess the associative utility of the SII and BMI for the clinical efficacy of acupuncture in patients with RHS–associated facial palsy, ROC curve analyses were performed (Table 4 and Figure 1). The discriminatory performance of each parameter, as well as their combined associative performance, is summarized in Table 4. SII demonstrated good discriminative ability, with an area under the ROC curve (AUC) of 0.839 (95% CI: 0.765–0.913, *p* < 0.001), indicating a notable association with treatment outcome. The optimal cut-off point for SII was determined to be 1262.935, at which the sensitivity and specificity for distinguishing non-response to acupuncture were 70.0 and 89.4%, respectively. This suggests that elevated SII levels may be indicative of poorer clinical response. In comparison, BMI exhibited a more modest discriminative capacity, with an AUC of 0.665 (95% CI: 0.561–0.769, *p* = 0.003). At the best cut-off value of 23.875, the sensitivity reached 80.0%, but the specificity was relatively lower at 54.1%. These results suggest that although BMI may provide some association with outcomes, its discriminatory power is limited when used alone. Notably, when SII and BMI were combined into a composite model, the AUC reached 0.840 (95% CI: 0.767–0.913, *p* < 0.001), with an improved sensitivity of 85.9% and specificity of 72.5%. Given that the increase in AUC compared with SII alone was minimal and not statistically validated, these findings suggest that integrating systemic inflammatory status with anthropometric measures may provide limited additional value in exploratory models; however, confirmation in larger, adequately powered cohorts is warranted. Together, these findings support the potential clinical relevance of SII as an independent biomarker associated with therapeutic outcomes in this population and suggest that its associative value may be strengthened when considered alongside BMI.

**Table 4:** The ROC analysis evaluating the effects of SII on the prediction of clinical efficacy of acupuncture for facial palsy in Ramsay-Hunt syndrome.

Variables	AUC	95% CI	Best cut-off value	Sensitivity (%)	Specificity (%)	*p* value
SII	0.839	0.765 ~ 0.913	1262.935	0.7	0.894	<0.001
BMI	0.665	0.561 ~ 0.769	23.875	0.8	0.541	0.003
Combined	0.84	0.767 ~ 0.913		0.859	0.725	<0.001

## Discussion

This study represents, to our knowledge, the first retrospective analysis to explore the potential of the systemic SII as a biomarker associated with acupuncture efficacy in the treatment of facial palsy secondary to RHS. Our findings demonstrate that SII is significantly elevated in patients who failed to achieve clinical improvement following acupuncture therapy. Furthermore, multivariate logistic regression identified SII as an independent factor negatively associated with therapeutic response, and ROC curve analysis confirmed its notable discriminative ability. These results highlight the potential role of SII as a clinically relevant indicator that may contribute to prognostic assessment and patient stratification in acupuncture-based interventions for RHS-associated facial paralysis.

From a hematological perspective, our results demonstrated that non-responders not only had significantly elevated neutrophil counts but also exhibited decreased lymphocyte counts, both of which are critical contributors (20) to the elevated SII values observed. As a composite index, SII reflects the balance between pro-inflammatory and immune-regulatory components (21, 22). Neutrophils are first-line responders in acute inflammation and are known to mediate tissue injury through degranulation and the release of reactive oxygen species, neutrophil extracellular traps (NETs), and pro-inflammatory cytokines such as interleukin-6 (IL-6) and tumor necrosis factor-alpha (TNF-α) (23–25). Their overactivation can exacerbate local inflammation, leading to prolonged nerve damage (26, 27). Conversely, lymphocytes, particularly T-helper and regulatory T cells, play a central role in resolving inflammation and orchestrating tissue repair mechanisms (28, 29). Reduced lymphocyte levels, as found in the non-responders, may be associated with a compromised adaptive immune response and impaired resolution of inflammation, which in turn may contribute to hinder nerve recovery and reduce the efficacy of acupuncture’s restorative functions.

The pathophysiological interplay between systemic inflammation and facial nerve injury in RHS offers a plausible explanation for the observed association between elevated SII and poor clinical outcomes. RHS is initiated by the reactivation of latent varicella-zoster virus (VZV) within the geniculate ganglion, resulting in an intense inflammatory response that causes demyelination and axonal degeneration of the facial nerve (1, 3, 30, 31). The magnitude and persistence of this immune-mediated injury are key determinants of the extent of nerve dysfunction and the potential for recovery (32). Inflammatory cytokines such as IL-1β and TNF-*α* not only recruit additional immune cells but also disrupt the blood-nerve barrier, thereby facilitating further neural damage (33, 34). In this context, a heightened SII may reflect a systemic inflammatory milieu that reinforces local pathology and impairs the neuroprotective and immunomodulatory mechanisms purportedly triggered by acupuncture. By promoting local circulation, regulating autonomic function, and modulating neuroimmune interactions, acupuncture theoretically aids in neural repair; however, this benefit may be significantly attenuated in the presence of systemic hyperinflammation.

An additional dimension of our study pertains to the role of BMI, which was initially found to be negatively correlated with acupuncture efficacy. Higher BMI has long been associated with chronic low-grade inflammation due to the metabolic activity of adipose tissue (35–37). Adipocytes and infiltrating macrophages in obese individuals secrete an array of pro-inflammatory mediators, including IL-6, TNF-*α*, and leptin, which contribute to elevated neutrophil levels and impaired lymphocyte function (38, 39). While BMI lost its statistical significance in the multivariate model, its initial association suggests that body composition may influence therapeutic response via inflammatory pathways, a relationship effectively captured and mediated by the SII. This highlights the broader value of SII in integrating anthropometric and immunological signals into a single, functionally relevant biomarker.

Our ROC analysis further suggests the potential prognostic relevance of SII. The high specificity (89.4%) at an optimal cut-off of 1262.935 suggests that elevated SII may help distinguish patients at risk of non-response to acupuncture. This threshold, derived from the maximum Youden index, represents the point of best balance between sensitivity and specificity, and although it showed good discriminatory performance in our cohort, its clinical applicability should be interpreted cautiously and requires validation in larger, prospective studies before routine adoption. While BMI alone exhibited modest discriminative capacity (AUC = 0.665), the combination of SII and BMI yielded a slight but limited improvement in discriminatory performance (AUC = 0.840), indicating a possible contribution of a multifactorial model in outcome assessment. Such an approach may have potential clinical utility in supporting treatment decisions by helping to identify patients who might benefit from early adjunctive interventions, such as antiviral therapy intensification, corticosteroids, or neuromodulation strategies, in addition to acupuncture.

On a mechanistic level, several molecular pathways may mediate the observed dampening of acupuncture efficacy under conditions of elevated systemic inflammation. Acupuncture is known to engage the hypothalamic–pituitary–adrenal (HPA) axis and modulate sympathetic and parasympathetic activity, leading to the suppression of pro-inflammatory cytokines and upregulation of anti-inflammatory mediators such as interleukin-10 (IL-10) (40–42). These effects are mediated in part by activation of the vagus nerve and the cholinergic anti-inflammatory pathway (43, 44). However, in a system overwhelmed by excessive inflammatory stimuli, these regulatory mechanisms may be functionally impaired. Moreover, systemic inflammation has been shown to induce endothelial dysfunction and microcirculatory impairment, which could limit the perfusion and oxygenation of injured nerve tissue (45), thereby restricting the biological benefits of acupuncture-induced vasodilation and metabolic modulation (46). These processes may collectively contribute to the observed reduction in acupuncture efficacy among patients with high SII.

In addition to its association with acupuncture outcomes, SII may also have broader relevance in guiding other therapeutic strategies for RHS. Antiviral agents and corticosteroids represent the cornerstone of standard treatment, but their efficacy is partly dependent on the host’s immunoinflammatory status. Elevated SII, reflecting increased systemic inflammation and impaired immune regulation, may compromise antiviral effectiveness and attenuate corticosteroid responsiveness by limiting resolution of inflammation. Conversely, lower SII values may indicate a more balanced immune environment, favoring better therapeutic outcomes. Importantly, acupuncture, by modulating local neuroimmune responses and potentially reducing systemic inflammatory burden, might exert complementary effects that enhance the efficacy of antiviral and corticosteroid therapy. Thus, SII not only serves as a potential biomarker for acupuncture responsiveness but may also help stratify patients who could benefit from intensified pharmacological regimens or early integration of adjunctive therapies. Future prospective studies are warranted to investigate these interactions and to clarify whether SII-guided integrative approaches can optimize therapeutic outcomes in RHS.

This study has several limitations. First, its retrospective, single-center design with a relatively small sample size and no *a priori* power calculation may introduce selection bias, limit causal inference, and reduce generalizability. Second, the natural course of RHS recovery was not fully accounted for, although we restricted inclusion to patients treated within 7 days of onset. Third, despite standardized acupuncture protocols, variation in manual stimulation may have introduced confounding. Fourth, although all patients received antivirals and corticosteroids under institutional protocols and their use was balanced between groups, residual confounding from these treatments cannot be excluded. Fifth, only a single baseline SII measurement was available, precluding evaluation of dynamic inflammatory changes. Future prospective, multicenter studies with larger, adequately powered cohorts and longitudinal biomarker tracking are needed to validate these findings and clarify mechanistic links between systemic inflammation and acupuncture responsiveness.

## Conclusion

In conclusion, this study suggests that SII may serve as a potential independent indicator of acupuncture efficacy in patients with RHS-induced facial paralysis. Elevated SII was associated with reduced likelihood of recovery, likely reflecting heightened inflammation that impedes neural repair. SII assessment could therefore aid clinical evaluation and support individualized treatment strategies. Although combining SII with BMI yielded only marginal improvement, it may still offer additional prognostic insight. These findings highlight the potential value of immunological profiling in guiding RHS management. Given the relatively small sample size, future prospective, multicenter studies with larger cohorts and longitudinal biomarker assessments are needed to validate these results.

## Data Availability

The original contributions presented in the study are included in the article/supplementary material, further inquiries can be directed to the corresponding author.
